# Minifragment plating of the fibula in unstable ankle fractures

**DOI:** 10.1007/s00402-022-04397-2

**Published:** 2022-02-28

**Authors:** D. Penning, C. A. L. Jonker, R. Buijsman, J. A. Halm, T. Schepers

**Affiliations:** 1 Trauma Unit Amsterdam UMC, Location AMC, Meibergdreef 9, 1105 AZ Amsterdam, The Netherlands; 2 Department of Traumasurgery, Tergooi MC, Van Riebeeckweg 212, 1213 XZ Hilversum, The Netherlands

**Keywords:** Distal fibula fracture, Minifragment, Small fragment, Ankle fracture, Fibula

## Abstract

**Introduction:**

Only 6.4–17% of the load is transmitted through the fibula when weight-bearing. Plate fixation of distal fibular fractures using minifragments (≤ 2.8 mm) could lead to similar reduction with less implant removal (IR) rates, compared to small-fragment plates (3.5 mm). We hypothesized that the use of minifragment plates is at least similar in unscheduled secondary surgery.

**Materials and methods:**

In this retrospective cohort study, all patients with surgically treated distal fibular fractures between October 2015 and March 2021 were included. Patients treated with plate fixation using minifragments and patients treated with small-fragment plates were compared regarding the following outcomes: secondary dislocation, malreduction, implant malposition, nonunion, surgical site infections (SSI) and IR.

**Results:**

Sixty-five patients (54.2%) received a minifragment implant (≤ 2.8 mm) and 55 patients (45.8%) received a small-fragment implant (3.5 mm). There were no patients needing secondary surgery in the minifragment group compared to 9 patients following fixation using small-fragment implants (3 with secondary dislocation, 5 with malreduction and 1 with malposition, *p* = 0.001). SSI rates were 3.1% for minifragment and 9.1% for small-fragment implants (*p* = 0.161). Implant removal was performed significantly less often following use of minifragment implants (17.8% and 53.2%, *p* < 0.001).

**Conclusions:**

In this cohort, minifragment plate fixation for distal fibular fractures is an adequate fixation method offering stable fixation with significant lower need for implant removal and comparable complications to small-fragment plates, although an adequately powered randomized controlled study is needed for implementation in a clinical setting.

**Level of evidence:**

Therapeutic, III.

## Introduction

The number of ankle fractures has increased over time, most likely due to a more active elderly population [1–3]. The number of ankle fractures peaks at the adolescent age for both genders and has a second peak in elderly female patients [3–5].

Most ankle fractures involve the fibula. Isolated fibula fractures are responsible for 55–70%, bimalleolar 4–20% and trimalleolar 10–11% of fractures around the ankle [2, 3, 6]. About half of the ankle fractures needs surgical stabilization because of instability and posterior or medial involvement [7].

The treatment of ankle fractures has changed over time and this evolution has been influenced by some of the four milestones in history—the introduction of anesthesia (1846), antisepsis (1865), radiography (1895) and antibiotics (1936–44) [8]. Regarding the fixation of the fibula, different implants are available, including intramedullary devices, such as intramedullary screws and specially designed nails, lag screws only, cerclage and plate fixation [9–12].

**Fig. 1:**
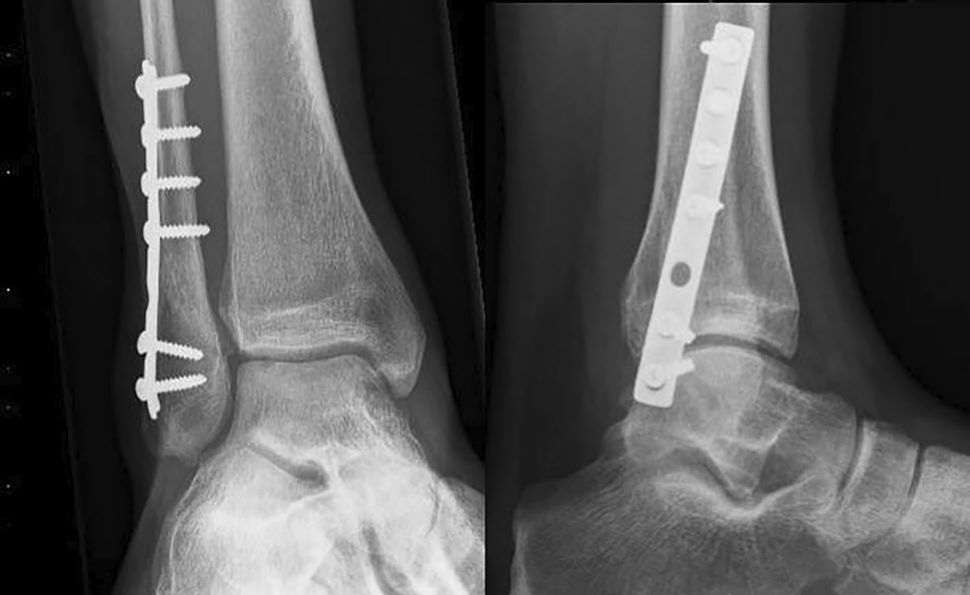
Postoperative image of distal fibular fixation using small fragment with anteroposterior view (left) and lateral view (right)

Plate fixation has traditionally been achieved with one-third tubular plates [13–15]. More recently, anatomically precontoured (locking compression plates; LCP) have become available [16, 17]. Biomechanically, a benefit for locking plate constructs was only shown in patients with severe osteoporosis. The downside of utilizing small fragment (3.5 mm screws, Fig. 1) locking plates might be an increase in wound complication rates (superficial and deep infection) and wound dehiscence [18, 19].

Takebe, Goh and later Calhoun and Wang showed that 6.4–17% of the load is transmitted through the fibula while standing [20–23]. This is determined by the position of the ankle, being lowest in a neutral position.This brings up the issue how much implant is needed to stabilize a fibular fracture.

**Fig. 2:**
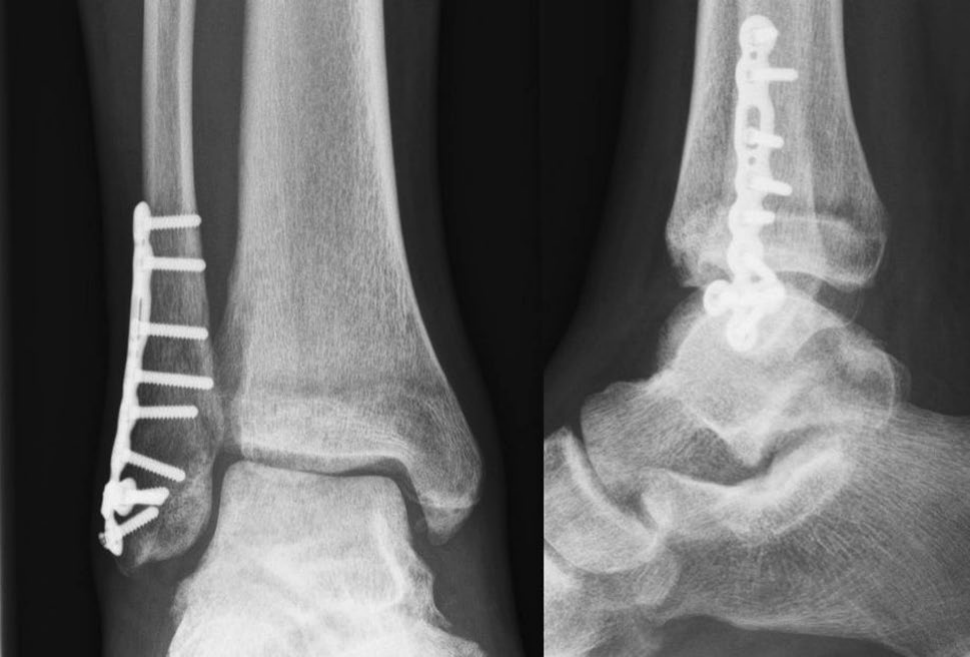
Postoperative image of distal fibular fixation using minifragment with anteroposterior view (left) and lateral view (right)

To reduce complication rates and need for implant removal, less bulky implants might be a solution. Only limited data are available on the use of minifragment plates (2.8 mm and smaller, Fig. 2) for fibular fractures. Bariteau et al. showed that two 2.4-mm lag-screws performed similar to one 3.5-mm lag-screw in a biomechanical fibula fracture model [24]. In addition, no significant difference in mean stiffness or mean load to failure between a 2.4-mm plate-screw construct and a 3.5-mm plate-screw construct was found. One clinical study, comparing patients (*n* = 28) with a fibula fracture treated with a small-fragment (one-third tubular plate) versus 16 minifragment plates, showed that minifragment plate use was safe [25].

We hypothesized that the use of minifragment plates is at least similar in maintaining reduction without increasing revision rates. In addition, the rate of wound complications and need for implant removal (IR) are expected to be lower in minifragment plates compared to small-fragment (3.5 mm) plates.

## Materials and methods

In this retrospective cohort study, all patients with surgically treated distal fibular fractures between October 2015 and March 2021 were included. Data were collected at a single level-1 trauma center in the Netherlands, using the codes used to register different types of surgery.

Letters for no-objection to use patient data were sent to eligible patients, and patient data were anonymized.

Inclusion criteria—ankle fracture with concomitant fracture of the fibula treated by open reduction and internal fixation with plate fixation.

Exclusion criteria—fractures of the tibial pilon, isolated medial malleolar fractures, nonoperative treatment, fixation with syndesmotic screws only, other fixation than plate fixation of the distal fibula and insufficient follow-up (FU).

The choice of which implant was opted for was left to the discretion of the surgeon.

Data collection was performed by three authors (DP, CJ and RB). Implant size was registered from the operative notes. Registered implants size was double checked by two authors (DP and TS) using available x-ray images.

If a patient had bilateral procedures, these were registered separately.

The collected patient and injury characteristics were age, gender, weight, American Society of Anesthesiologists (ASA) class, active smoker, open or closed fracture classified according to Gustilo, and fracture type according to the AO/Weber classification.

Surgical characteristics were implant size categorized in minifragment plates (2.0, 2.4, 2.7 or 2.8 mm) or small-fragment plates (3.5 mm), external fixation prior to definitive open reduction and internal fixation (ORIF).

### Outcome

The primary outcome was the need for secondary surgery following initial fixation. This included secondary surgery for secondary dislocation, malreduction, implant malposition and nonunion. Secondary dislocation was scored when the initial reduction was adequate, but the fracture secondary dislocated after surgery. Malreduction was scored when inadequate fracture reduction was noted on the intra-operative image-intensifier images in retrospect. Malposition was defined as inadequate positioning of the implant (plate or screw) directly following internal fixation.

A nonunion was defined as insufficient bone union at six months or when the patient was symptomatic with an incomplete fracture healing on a conventional radiograph [26]. Secondary outcomes were the incidence of surgical site infections (SSI) and implant removal (IR).

Follow-up (FU) visits were protocolled at 2 weeks, 6 weeks and 3 months following discharge and patients were instructed to contact the hospital in case of wound complications.

Any revision surgery related to the implants or fracture reduction and in case of deep SSI were recorded.

Minimal FU required for complication registration was 3 months for malreduction, malposition and SSI, 6 months for secondary dislocation and nonunion and 12 months for IR.

### Statistical analysis

Statistical analyses were performed using IBM SPSS Version 26.0.0.1.

Continuous variables were tested for normality using the Kolmogorov–Smirnov test and Shapiro–Wilk test. The continuous variables were normally distributed when the significance was > 0.05.

Pearson chi-squared was used to compare categorical variables and a Students’s *T* test was used to compare ordinal and normally distributed continuous variables. Continuous variables without normal distribution were analyzed using a Mann–Whitney *U* test.

Statistical significance was defined as *p* < 0.05. Due to the hospital wide implementation of the electronic medical records in October 2015, all data were collected from this date onwards.

## Results

A total of 268 procedures were reviewed following the initial search. Based on the inclusion and exclusion criteria, 120 procedures were included for analysis.

Characteristics of this cohort are shown in Table 1, which is categorized in two groups based on the dimensions of used implants: ≤ 2.8 mm and 3.5 mm. Sixty-five patients (54.2%) received a minifragment implant (≤ 2.8 mm) and 55 patients (45.8%) received a small-fragment implant (3.5 mm). Both the variables age at surgery and weight were normally distributed (Kolmogorov–Smirnov test, respectively, 0.99 and 0.94).

**Table 1:** Patient characteristics

	Implant size		
	Minifragment	Small fragment	Significance (*p*)
*n* (%)	65 (54.2%)	55(45.8%)	
Follow-up			
< 3 months, *n* (%)	0 (0%)	0 (0%)	
3–6 months, *n* (%)	13 (20.0%)	3 (5.5%)	
6–12 months, *n* (%)	20 (30.8%)	8 (14.5%)	
> 12 months, *n* (%)	45 (69.2%)	47 (85.5%)	
Age, mean (SD)	44.8 (17.5)	47.6 (17.6)	0.809
Gender M:F (% male)	39:26 (60.0%)	23:32 (41.8%)	**0.047**
Active smoker, *n* (%)	11 (16.9%)	15 (27.3%)	0.200
ASA class (%)			
1/2	60 (92.3%)	49 (89.1%)	0.521
3/4	5 (7.7%)	6 (10.9%)	
Weight, mean kg (SD)	81.4 (15.6)	79.6 (13.5)	
Missing (*n*)	5	2	0.502
Fracture Weber classification			
Weber A	2 (3.1%)	0 (0%)	0.413
Weber B	40 (61.5%)	36 (65.5%)	
Weber C	23 (35.4%)	19 (34.5%)	
Open fracture *n* (%)	6 (9.2%)	8 (14.5%)	0.366
Gustilo 1	0 (0.0%)	1 (1.8%)	0.556
Gustilo 2	4 (6.2%)	3 (5.5%)	
Gustilo 3	2 (3.1%)	2 (3.6%)	
Missing (*n*)	0 (0.0%)	1 (1.8%)	
External fixation prior to ORIF, *n* (%)	9 (13.8%)	5 (9.1%)	0.419
Implant complications, *n* (%)	0 (0%)	9 (16.4%)	**0.001**
Secondary dislocation	0 (0%)	3 (5.8%)	0.079
Malreduction	0 (0%)	5 (9.1%)	**0.013**
Nonunion	0 (0%)	0 (0%)	
Malposition	0 (0%)	1 (1.8%)	0.275
SSI	2 (3.1%)	5 (9.1%)	0.161
Plate removal	8 (17.8%)	25 (53.2%)	**0.001**
Syndesmotic screw removal	1 (2.2%)	0 (0%)	0.437

Bold values indicate a significant difference (*p* < 0.05)

Age and weight did not differ significantly, however, significantly more male patients were treated using minifragment plating (*p* = 0.047). The location of the fracture, using the Weber classification, did not vary significantly between the two groups and neither did the percentage of open fractures, Gustilo grade and the use of external fixation prior to ORIF. In one case, the fracture was described as open but no Gustilo grade was available. With the existing information in the medical record, it was not possible to classify this fracture according to Gustilo.

All patients had a minimal FU of 3 months. Thirteen procedures using minifragment implants had a FU time of 3–6 months (20.0%) compared to 3 procedures using small-fragment implants (5.5%).

Twenty procedures using minifragment implants had a FU time of 6–12 months (30.8%) compared to 8 procedures using small-fragment implants (14.5%). FU of more than 12 months was seen in 45 (69.2%) procedures using minifragment implants and 47 (85.5%) procedures using small-fragment implants. Mean FU of the group with sufficient FU to score IR was 38 months following fixation using minifragments and 63 months following fixation using small-fragment implants.

Following fixation with minifragment plates, no patients developed implant complications including secondary dislocation, malreduction, malposition and nonunion. Following fixation with small-fragment plates, nine patients developed implant complications (16.4%). This was a significant difference (*p* = 0.001). Secondary dislocation was seen three times following fixation with small-fragments (5.8%), while no patients developed secondary dislocation following fixation using minifragments (*p* = 0.076). In this group, no patients showed malreduction following fixation with minifragment plates compared to 5 patients following fixation using small-fragment plates(9.1%, *p* = 0.013).

Malposition rate was not significantly different between the two groups, with one patient with malposition of a screw in the small-fragment plate (1.8%). No nonunions were encountered in this cohort. There was no significant difference in SSI rate between minifragment plating (2 patients, 3.1%) and patients treated with small-fragment plates (5 patients, 9.1%).

Implant removal of the plate was performed significantly less following treatment with minifragment implants (*p* = 0.001). Mean time to IR was 18 months following fixation using small-fragment implants.

## Discussion

There were significantly more patients with implant complications needing secondary surgery following plate fixation using 3.5 mm implants. This might have been partially attributed to the fact that some patients were referred to our academic center following primary fixation elsewhere. However, this indicates that there is no reason to assume minifragment plate fixation offers inferior stabilization compared to conventional plate fixation. This is in accordance with the biomechanical study by Bariteau et al. and the clinical study by Gentile et al. who described stable fixation using minifragment plates [24, 25]. Our study included a larger clinical cohort to support this conclusion.

As nonunion may be related to incomplete reduction or loss of reduction in complex fractures, this therefore strengthens the conclusion that fixation using minifragments is non-inferior to small-fragment implants [27].

One of the secondary aims of this study was to investigate whether the less bulky minifragment plates would require less IR than the current standard, the small-fragment plates. There was significantly less plate removal following the use of minifragments (12.3 vs 45.5 *p* = 0.001) in patients with a minimal follow-up of 1 year. With infection ranging from 8 to 20% for implant removal, IR is an on demand procedure and is most frequently performed following local complaints of the implant. This may indicate that minifragment plating leads to less local complaints [28, 29].

In this cohort, there were more patients with a FU of less than 12 months in the minifragment group than in the small-fragment group because minifragment plates have been used more in recent years than at the start of data collection in 2015. IR rates are expected to be higher with longer FU. Therefore, some of the patients with short FU may still need IR in the future, although mean FU time in both groups we used to compare IR rates were longer than the mean time to IR (38 months following minifragment fixation and 63 months following small-fragment fixation). Gentile et al. described a mean cost of $400.77 for conventional plating group versus $1528.25 in the minifragment group [25]. Their conclusion was that this price would be easily justified when a decrease in hardware removal would be demonstrated. In this cohort, two types of minifragment plates were used. Our estimated average costs for the minifragment plates were 531 euro, compared to 227 euro for the small-fragment plates, making the minifragment plates 304 euro more expensive.

Additionally, mean costs for IR will be lower with lower rates of IR. In our center, costs for either complete or partial implant removal were scheduled at 1560 euro. These costs were made for 20.0% (including 1 patient in which only the syndesmostic screw was removed and the plate left in situ) of the patients treated with minifragments and 53.2% of the patients treated with small-fragments (difference 33.2%). Therefore, average removal costs are roughly scheduled at 312 euro per patient treated with minifragment plates and 830 euro per patient treated with small-fragment plates. This results in a difference of 518 euro per patient on average, making the minifragment plates cost effective in this cohort. Furthermore, the use of minifragment implants reduces the burden of possible complications following implant removal as a result of lower removal rates [30].

Infection rates following surgical treatment of ankle fractures usually range from 1.4 to 5.5% [18, 31, 32]. In our cohort, comparable infection rates have been observed, although the rate in the small-fragment group was slightly higher (9.1%). However, the ankle fractures and the patient characteristics presented in this level-1 trauma center may not be a good representation as more severely injured patients with possibly higher rates of comorbidity were treated at our center.

In the current study, we did not look specifically at patients with severe comorbidity such as overweight and diabetes. Although based on the literature, it might be of benefit to use more rigid fixation (e.g., 3.5 mm plates) in such cases, especially in patients suffering from diabetes with polyneuropathy [33, 34].

### Strengths and limitations

This cohort can be considered relatively small, although this cohort is larger than the cohort used in previous studies. Furthermore, not all patients have completed a follow-up of more than a year, but as discussed earlier, we believe that the amount of patients with a minimal follow-up of one year is enough for the indication that smaller fragments will lead to less implant removal.

This cohort had a higher percentage of male patients treated with minifragment plates. This is most likely attributed to the retrospective nature of this study. However, we do not believe this would change the beneficial outcome toward minifragment plates. Furthermore, implant removal is performed more frequently in male patients [35].

## Conclusion

In this cohort, minifragment plate fixation for distal fibular fractures is an adequate fixation method offering stable fixation with significant lower need for implant removal and comparable complications to small-fragment plates, although an adequately powered randomized controlled study is needed for implementation in a clinical setting.
